# Tendon split lengthening technique for flexor hallucis longus tendon rupture

**DOI:** 10.1186/s13018-017-0668-y

**Published:** 2017-11-06

**Authors:** Jae Yong Park, Chenyu Wang, Hee Dong Kim, Hyong Nyun Kim

**Affiliations:** 1 Department of Orthopedic Surgery, Hallym Sacred Heart Hospital, Hallym University College of Medicine, Anyang, South Korea; 2 Department of Orthopedic Surgery, Kangnam Sacred Heart Hospital, Hallym University College of Medicine, 948-1, Dalim-1dong, Youngdeungpo-gu, Seoul, 150-950 South Korea

**Keywords:** Great toe, Flexor hallucis longus, Tendon rupture, Tendon defect, Split tendon lengthening

## Abstract

**Background:**

Flexor hallucis longus (FHL) tendon rupture is a challenging injury to lead with clawing of the great toe when the FHL tendon is repaired too tight. When the diagnosis is delayed, the tendon ends may not be opposable because of contracture or poor tendon tissue.

**Methods:**

A technique to reconstruct FHL tendon rupture without a free tendon graft is described. A split tendon lengthening is performed at the midfoot around the knot of Henry. Ankle block anesthesia is used to allow the patient’s active movement of the interphalangeal (IP) joint to determine the appropriate length of the reconstructed tendon for maintaining balance and preventing the tendon from being too tight or too loose. Between May 2012 and September 2015, five patients with a total rupture of the FHL tendon, having tendon defect distal to the knot of Henry, were treated with split tendon lengthening.

**Results:**

Four patients could actively plantarflex the great toe IP joint. One patient who was operated under spinal anesthesia could not actively plantarflex the great toe IP joint, but did not have extension deformity and did not want another procedure. The mean American Orthopedic Foot and Ankle Society (AOFAS) score at a mean follow-up of 44 months was 92 points (range, 80–100).

**Conclusions:**

This technique is described to overcome the difficulty of reconstructing the FHL tendon with tendon defect. The tendon defect could be repaired after split tendon lengthening without a free tendon graft.

## Background

The flexor hallucis longus (FHL) tendon plantarflexes the distal phalanx of the great toe generating the last push-off power for walking, running, and jumping [1]. The FHL tendon is most commonly injured by laceration when the patient steps on sharp objects, such as broken glass while running or walking bare foot [2–4]. The FHL tendon is at risk during hallux surgery, such as Akin osteotomy, metatarsal osteotomy, or soft tissue release, because it passes close to the plantar aspect of the proximal phalanx and the metatarsal head [5, 6]. Closed traumatic rupture is also possible by a forceful dorsiflexion of the great toe [7–10]. Total rupture of the FHL tendon distal to the knot of Henry, the fibrous slip connecting the FHL and the FDL, can cause loss of push-off strength and later cause hyperextension deformity of the IP joint [3]. Thompson et al. [11] described a patient who complained that the dorsum of her great toe rubbed against the toe box of her shoe because of an extension deformity at the IP joint.

Open injury or closed isolated traumatic injury can be detected early, but when it is associated with more severe injury, such as fracture, or when the tendon is ruptured during hallux surgery, it may be overlooked because the patients may not complain of specific pain along the FHL tendon or loss of the great toe interphalangeal (IP) joint motion, until the more severe pain from a fracture or osteotomy subsides [1, 11–13]. Diagnosis of FHL tendon rupture may be delayed, and degenerative changes may be present both proximally and distally. The tendon ends may not be opposable because of contracture or poor tendon tissue. In cases of tendon retraction or defects, direct repair may cause clawing of the great toe due to tightening of the tendon, which may be more problematic during walking than the loss of IP joint active plantarflexion and the push-off strength [12]. When the FHL tendon is repaired too tight, there is a high possibility of re-rupture during walking, because walking gives passive dorsiflexion of the metatarsophalangeal (MTP) joint imposing significant amount of traction force at the repaired end. Free tendon graft is recommended for the FHL tendon rupture with tendon defect [1].

A technique to reconstruct FHL tendon rupture without a free tendon graft is described.

## Methods

### Patients

Five patients with a total rupture of the FHL tendon, having tendon defect distal to the knot of Henry and the fibrous slip, were treated with split tendon lengthening between May 2012 and September 2015. Two patients had laceration on the great toe, and primary repair was initially performed at the local hospitals. The patients were referred to our hospital when the loss of active flexion at the great toe IP joint was detected. Other two patients had dislocation of the IP joint, and closed reduction with trans-articular fixation was performed. FHL tendon rupture was detected 6 weeks later, when K-wire was removed. The last one had a history of IP joint dislocation, which was reduced at a local hospital 2 months ago. The patient had extension deformity at the IP joint and complained of the big toe rubbing against the toe box inside his shoe. The mean duration of time from the initial injury to FHL reconstruction surgery was 6.2 weeks. The mean defect size was 18.4 mm.

### Surgical technique

**Fig. 1:**
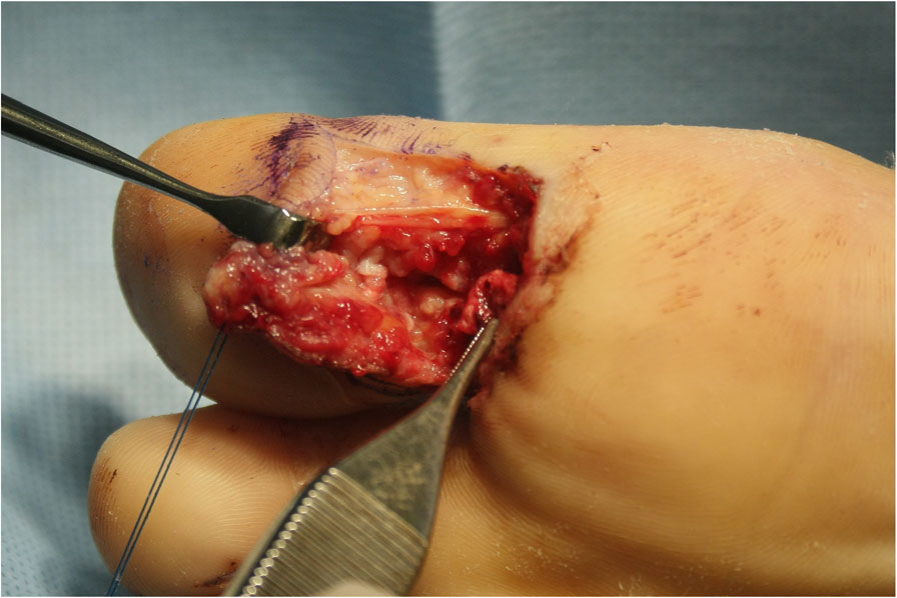
Degenerative tendon tissue was excised at the proximal and the distal ends of the rutpured FHL tendon and the defect size was measured between the both ends of the tendon

Under ankle block anesthesia, the patient was placed in a supine position with a pneumatic tourniquet prepared around the calf for later use during tendon length determining procedure. After routine sterile drape was placed, a rubber tourniquet was applied around the ankle. In cases of lacerated rupture, when the proximal end of the ruptured tendon could not be found because of retraction, incision was extended proximally through the medial or lateral side of the first metatarsal head longitudinally to avoid injury to the fat pad and the neurovascular bundle underneath the first metatarsal head. Degenerative tendon tissue was excised at the proximal and the distal ends of the ruptured FHL tendon, and the defect size was measured between both ends of the tendon, with the ankle joint in plantigrade neutral position and with the first MTP joint and the IP joint in neutral 0 degree position (Fig. 1).

**Fig. 2:**
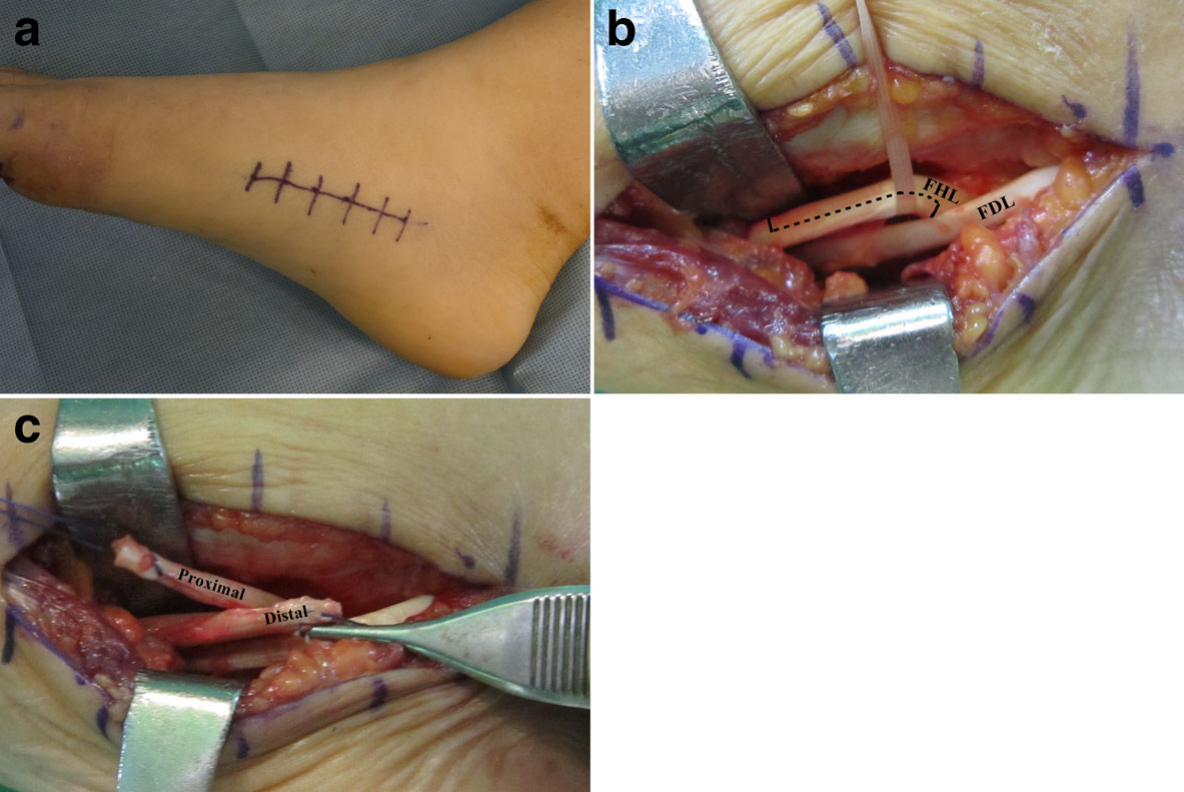
**a** In the midfoot, a longitudinal incision was made along the upper border of abductor hallucis muscle. **b** The FHL and FDL tendon were identified at the knot of Henry. A split incision (dotted line) was started at the knot of Henry and advanced distally 1.5 cm longer than the measured size of the tendon defect to divide FHL longitudinally into half. **c** Before the vertical cut at the end of the split incision, the whip stitch was made at both ends of the split tendon to secure the tendon from retracting proximally. FHL flexor hallucis longus, FDL flexor digitorum longus.

**Fig. 3:**
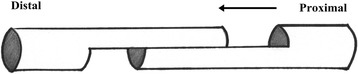
The vertical cut at the end of the split incision made the distal part of the FHL tendon move distally for lengthening

**Fig. 4:**
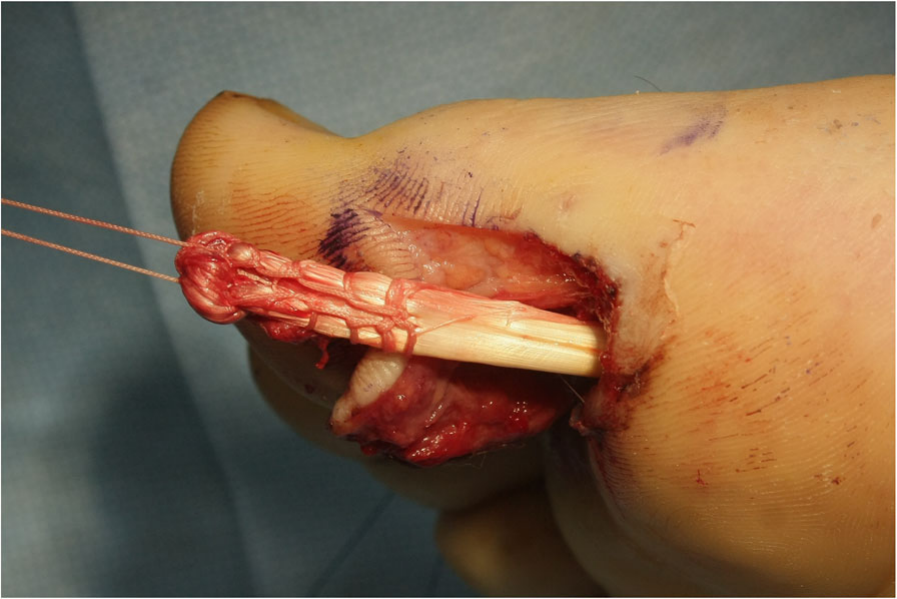
The whip stitch was made at the distal end of the ruptured tendon and was pulled down distally

**Fig. 5:**
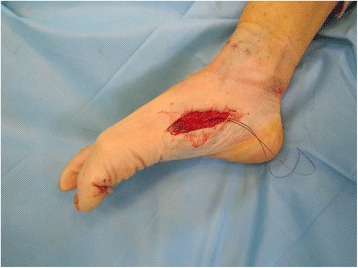
The length of the FHL tendon was determined at the midfoot where FHL was split. Overlap area of the split tendon was temporarily sutured in neutral position of the joints and was tested for balance with patient’s active movement. With the patient in ankle block anesthesia, the great toe could be actively moved by the patient and the length of the tendon could be adjusted when it was too tight or too loose

In the midfoot, a longitudinal incision was made along the upper border of abductor hallucis muscle (Fig. 2a). The FHL and flexor digitorum longus (FDL) tendons were identified at the knot of Henry. The fibrous slip connecting the FHL and FDL was divided and released. A split incision was made to divide FHL longitudinally into half (Fig. 2b) [14]. The split incision was started at the knot of Henry and advanced distally 1.5 cm longer than the measured size of the tendon defect to facilitate later overlap for suturing after lengthening of the split tendon. Before the vertical cut at the end of the split incision, the whip stitch was made at both ends of the split tendon to secure the tendon from retracting proximally (Fig. 2c). The vertical cut at the end of the split incision made the distal part of the FHL tendon move distally for lengthening (Fig. 3). The whip stitch was made at the distal end of the ruptured tendon and was pulled down distally (Fig. 4). Care was taken to hold the whip stitch at both ends and not to pull the tendon entirely out of the foot. If it was pulled out from the foot, it would be difficult to reinsert the tendon to its anatomical position, unless by means of open surgery. In two cases, there was some fibrous connection underneath the MTP joint that we had to release in order to pull down the tendon distally. When the distal stump of the ruptured FHL was healthy and long enough for a secure repair, end-to-end direct repair was possible. However, when the distal stump was degenerated or was too short, a bone hole was made to insert the distal end of the split FHL tendon. By using the pull-out technique, the two ends of the whip stitch were pulled out through the bone hole and were sutured on the dorsum of the distal phalanx. Finally, the length of the FHL tendon was determined at the midfoot, where the FHL was split. When the tendon is reconstructed too tight, it can cause clawing of the great toe and limit dorsiflexion, which can be problematic during walking. When it is too loose, a patient is not able to plantarflex the great toe IP joint, and it does not provide enough push-off strength during walking. To measure the appropriate length of the FHL tendon, we used ankle block anesthesia instead of general or spinal anesthesia to let the patient actively move the great toe to test balance. Overlap area of the split tendon was temporarily sutured in neutral position of the ankle, MTP joint, and IP joint and was tested for balance with patient’s movement (Fig. 5). The length was adjusted when the tendon was too tight or loose. During this procedure, pneumatic tourniquet was inflated at the calf, and the rubber tourniquet applied around the ankle was released, because we found that compression around the ankle plantarflexed the great toe making the tendon too tight to measure the balance. Patients could withstand 250 mmHg of pneumatic pressure around the calf even with ankle block anesthesia. In a single case with hyperextension deformity at IP joint, we repaired the plantar plate before the FHL reconstruction.

### Rehabilitation protocol

Postoperatively, short leg cast was applied for 3 weeks with a window made on the plantar area of the great toe to allow active plantarflexion exercise but to limit extension. Active extension exercise was encouraged after cast removal for the next 3 weeks. Weightbearing was allowed in a functional brace at 6 weeks postoperatively. Patients were invited to a final follow-up office visit for a detailed evaluation for the study.

## Results

One patient with laceration could not actively plantarflex the great toe IP joint even on the last follow-up visit at postoperative 63 months. The patient did not have extension deformity of the IP joint and did not want another procedure to restore IP joint flexion. This patient was operated under spinal anesthesia, and the tendon length and the balance were not tested by patient’s active movement of the great toe. The tendon length may have been too long making the tendon too loose. In this patient, the small sesamoid bone was attached to the distal stump. The sesamoid bone was removed because there was not enough remnant tissue for the repair. This sesamoid bone could have worked as a fulcrum to flex the IP joint; removing it may have affected the active IP joint plantarflexion. Other four patients could actively plantarflex the great toe IP joint, and the power of the great toe was equivalent to that of the unaffected side. Three of the patients were very satisfied with the results, and one was satisfied with the results. The mean American Orthopedic Foot and Ankle Society (AOFAS) score at a mean of 44 months was 92 points (range, 80–100 points; Table 1).

**Table 1:** Patients’ demographics and the results

No.	Age	Sex	Injury pattern	Duration	Tendon size defect (mm)	FU (months)	Active IP joint plantarflexion	AOFAS score
1	35	M	Laceration	6 weeks	18	63	Impossible	80
2	21	M	Dislocation	6 weeks	18	54	Possible	95
3	24	M	Laceration	4 weeks	21	53	Possible	95
4	54	M	Dislocation	8 weeks	16	26	Possible	100
5	40	M	Dislocation	7 weeks	20	24	Possible	90

## Discussion

In the literature, there are many case reports of FHL tendon rupture, and in most reports, even after meticulous surgery and repair of the tendon, active plantarflexion of the IP joint of the great toe remains disturbed [1, 3]. The reason for this loss of IP joint active plantarflexion is not well described. Spontaneous re-rupture could have happened, or the tendon could have been reconstructed too loose, not imposing enough strength on the distal phalanx while it is contracting [1]. Split lengthening of the FHL tendon enables excision of the degenerated tendon tissue because there is sufficient length of healthy tendon for reconstruction after lengthening. Repair with the healthy tendon lowers the re-rupture rate [15]. Furthermore, split lengthening of the FHL tendon around the knot of Henry enables determination of the appropriate length of the tendon to restore balance. With the patient in ankle block anesthesia, the great toe could be actively moved by the patient, and the length could be adjusted when it is too tight or loose. This enables more aggressive postoperative rehabilitation for better function [16].

Small number of cases is the limitation of this study. There are some case reports of successful conservative treatment after closed traumatic rupture of the FHL tendon. However, if the suturing of the tendon is not done, there is a theoretical chance of developing hyperextension deformity [17].

The FHL tendon lengthening technique at the midfoot around the knot of Henry for reconstruction and the technique to determine the appropriate length of the reconstructed tendon by the patient’s active movement of the joint will help surgeons treat this injury.

## Conclusions

This technique is described to overcome the difficulty of reconstructing the FHL tendon with tendon defect. The tendon defect could be repaired after split tendon lengthening without a free tendon graft.

## Abbreviations

AOFAS: American Orthopedic Foot and Ankle Society
FDL: Flexor digitorum longus
FHL: Flexor hallucis longus
FU: Follow-up
IP: Interphalangeal
MTP: Metatarsophalangeal
